# High-throughput analysis of anthocyanins in horticultural crops using probe electrospray ionization tandem mass spectrometry (PESI/MS/MS)

**DOI:** 10.1093/hr/uhad039

**Published:** 2023-02-28

**Authors:** Misaki Ishibashi, Kei Zaitsu, Ikue Yoshikawa, Shungo Otagaki, Shogo Matsumoto, Akira Oikawa, Katsuhiro Shiratake

**Affiliations:** Graduate School of Bioagricultural Sciences, Nagoya University, Chikusa, Nagoya, Aichi 464-8601, Japan; Graduate School of Agriculture, Kyoto University, Sakyo, Kyoto 606-8502, Japan; Faculty of Biology-Oriented Science and Technology, Kindai University, Nishimitani, Kinokawa, Wakayama 649-6493, Japan; Graduate School of Bioagricultural Sciences, Nagoya University, Chikusa, Nagoya, Aichi 464-8601, Japan; Graduate School of Bioagricultural Sciences, Nagoya University, Chikusa, Nagoya, Aichi 464-8601, Japan; Faculty of Agriculture, Meijo University, Tenpaku, Nagoya, Aichi 468-8502, Japan; Graduate School of Bioagricultural Sciences, Nagoya University, Chikusa, Nagoya, Aichi 464-8601, Japan; Graduate School of Agriculture, Kyoto University, Sakyo, Kyoto 606-8502, Japan; Graduate School of Bioagricultural Sciences, Nagoya University, Chikusa, Nagoya, Aichi 464-8601, Japan

## Abstract

Plant secondary metabolites exhibit various horticultural traits. Simple and rapid analysis methods for evaluating these metabolites are in demand in breeding and consumer markets dealing with horticultural crops. We applied probe electrospray ionization (PESI) to evaluate secondary metabolite levels in horticultural crops. PESI does not require pre-treatment and separation of samples, which makes it suitable for high-throughput analysis. In this study, we targeted anthocyanins, one of the primary pigments in horticultural crops. Eighty-one anthocyanins were detected in approximately 3 minutes in the selected reaction-monitoring mode. Tandem mass spectrometry (MS/MS) could adequately distinguish between the fragments of anthocyanins and flavonols. Probe sampling, an intuitive method of sticking a probe directly to the sample, could detect anthocyanins qualitatively on a micro-area scale, such as achenes and receptacles in strawberry fruit. Our results suggest that PESI/MS/MS can be a powerful tool to characterize the profile of anthocyanins and compare their content among cultivars.

## Introduction

Horticultural crops contain various secondary metabolites representing unique traits such as color, aroma, taste, and functionality [1–3]. Anthocyanins are important metabolites that reflect unique colors [2]. The chemical structures of anthocyanins are glycosides of anthocyanidins, and six major basic structures are observed [4]. These compounds show various colors, except green, based on substituents at the B-ring, local pH, or both [2]. Anthocyanins act as part of the stress defense function of crops, and also contribute to human health by protecting against cardiovascular diseases and cancer [2, 3, 5, 6]. It is important to understand anthocyanin profiles rapidly and spatially to enhance anthocyanin accumulation while breeding horticultural crops or anthocyanin intake while selecting horticultural products for consumption.

High-performance liquid chromatography (HPLC) and mass spectrometry (MS) were the main techniques used in previous anthocyanin analyses [4, 7, 8]. These are highly accurate but require many complicated steps and considerable time for preliminary treatment of plant samples and separation of anthocyanins. Ambient MS approach is one of simple analytical methods to solve these problems. Ambient MS create and analyze ions in various samples from plants and humans under ambient conditions [9]. For example, ambient MS techniques, such as desorption electrospray ionization (DESI) or direct analysis in real time (DART), were implemented for high-throughput analysis in plant and horticultural research [10–12]. Low-temperature plasma, electrospray laser desorption ionization, and dielectric barrier discharge ionization are also utilized in plant sciences and are suitable for imaging or single-cell analysis at the two- or three-dimensional stage [13–15].

Probe electrospray ionization tandem mass spectrometry (PESI/MS/MS), used in this study, is an ambient MS approach. PESI was developed by Hiraoka et al. [16, 17]. The ionization mechanism of PESI involves electrospray ionization (ESI) using a probe needle with a tip diameter of approximately 700 nm. A high voltage is applied to the probe, and the adherent compounds at the probe tip become ionizable. A small quantity of compounds is sufficient as the technique prevents loss during pre-treatment step [18]. As ionization is manageable even in a high-salt environment [19], it can also be applied to plant samples. Probes are available in various shapes and materials [17, 20, 21], and the probe used in this study is based on the design of an acupuncture needle and has reasonable portability and moderate robustness. Additionally, tandem mass spectrometry (MS/MS) improved the selectivity of the target compounds in the presence of matrix [18]. PESI/MS/MS has been used in biomedical engineering and forensic medicine research [22–25]. This approach may be applicable to horticulture crops, having complicated shapes and metabolites with similar structures such as anthocyanins.

In this study, we considered the application of PESI/MS/MS to horticultural crops, for the first time, and detected 81 anthocyanins associated with the structures of aglycones and glycosides. These anthocyanins have been assessed widely in horticultural fruits, vegetables, and flowers with little difference compared to using previous instruments like HPLC-based MS [4, 7, 26]. Thus, PESI/MS/MS could be used for high-throughput screening of anthocyanins in 16 species or cultivars and to develop a straightforward method for detecting molecular species qualitatively in micro-area organs.

## Results and discussion

### Detection of anthocyanins and discrimination from flavonols using PESI/MS/MS

First, we considered the conditions for measuring anthocyanins using PESI/MS/MS. Specifically, we evaluated and determined the cleavage conditions of the targeted compounds using chemical standards in product ion scan mode. In this study, we targeted major anthocyanins based on six basic structures, i.e. cyanidin, delphinidin, malvidin, pelargonidin, peonidin, and petunidin [2]. Each precursor ion was searched against ESI-QQ spectral data from the MassBank spectral database [27].

Anthocyanin has a positive charge in solution ([M]+). Therefore, we measured all samples in positive ion mode during this study. Generally, flavonol can be ionized efficiently in [M-H]^−^ state [8]. However, PESI/MS/MS also detect flavonols as [M + H]^+^ with the same *m/z* sizes to anthocyanins, because of the lack of separators, such as those used in LC–MS. We challenged the limited cleavage of fragments by increasing the collision energy in the range of −50 to −70 V for distinguishing between anthocyanins and flavonols. Specific product ions were identified in anthocyanins and flavonols respectively, e.g. *m/z* 125 only in delphinidin 3-hexoside (Del-3-Hex) and *m/z* 137 only in quercetin 3-hexoside (Que-3-Hex) (yellow arrows in Supplementary Fig. S1). In a few cases, the ion intensity of specific product ions was lower than that of aglycon ions, e.g. *m/z* 125 compared to 303 in Del-3-Hex (Supplementary Fig. S1). However, we prioritized specificity in distinguishing between major anthocyanins and flavonols.

Similarly, we also compared four other anthocyanins (cyanidin, pelargonidin, peonidin, and petunidin) and coordinated flavonols (data not shown). A similar pattern of cleavage was observed in DESI-MS [12], where pelargonidin 3-glucoside was cleaved into tiny pieces while passing it through MS^3^. Malvidin did not correspond to any flavonol in the MassBank database search results.

As another limitation, we could not distinguish several sugar structures using this method, e.g. the sugar structures of glucoside and galactoside. In this study, we made the notation for four main glycosides, i.e. hexoside (Hex), pentoside (Pen), rutinoside (Rut), and di-hexoside. Hexoside was selected as the main component of glycoside [4]. Rutinoside was selected because it is observed often in tree fruits and berries [28, 29]. The other glycosides were based on previous studies using HPLC-ESI-MS/MS [4, 7, 30–37]. The specific product ions in previous paragraphs were assigned to anthocyanins with four main glycosides. In contrast, other large glycosides were designated aglycone-sized ions based on detection sensitivity. In total, we could discriminate the five types of major anthocyanins from coordinated flavonols, and determined the selected reaction monitoring (SRM) transition for 81 anthocyanins and 5 flavonols (Table 1).

**Table 1:** Conditions of selected reaction monitoring (SRM) transition for anthocyanin and flavonol using PESI/MS/MS.

Compound	Abbreviation	Precursor ion^a^ (*m/z*)	Product ion^a^ (*m/z*)	Collision energy (V)	Chemical standard^b^
***Cyanidin***					
Cyanidin 3-glucoside	Cya-3-Hex	449.2	241.2	−60	+
Cyanidin 3-arabinoside	Cya-3-Pen	419.2	241.2	−60	−
Cyanidin 3-rutinoside	Cya-3-Rut	595.3	241.2	−70	+
Cyanidin 3,5-diglucoside	Cya-3-Hex,-5-Hex	611.2	241.2	−70	+
Cyanidin 3-(6″-malonyl)-glucoside	Cya-3-6″-Ma-Hex	535.2	287.2	−20	−
Cyanidin 3-(6″-acetyl)-glucoside	Cya-3-6”-Ac-Hex	491.2	287.2	−20	−
Cyanidin 3-(sinapoyl)-diglucoside-5-glucoside	Cya-3-Si-Hex-Hex,-5-Hex	979.2	287.2	−50	−
Cyanidin 3-(sinapoyl)(feruloyl)-diglucoside-5-glucoside	Cya-3-Si-Hex-Fe-Hex,-5-Hex	1155.2	287.2	−50	−
Cyanidin 3-(sinapoyl)(sinapoyl)-diglucoside-5-glucoside	Cya-3-Si-Hex-Si-Hex-,-5-Hex	1185.2	287.2	−50	−
Cyanidin 3–6″-malonyl-laminaribioside	Cya-3-6”-Mn-Hex-Hex	697.2	287.2	−30	−
Cyanidin 3-diglucoside-5-glucoside	Cya-3-Hex-Hex,-5Hex	773.2	287.2	−30	−
Cyanidin 3-(sinapoyl)-glucoside-5-glucoside	Cya-3-Si-Hex,-5Hex	817.2	287.2	−50	−
Cyanidin 3-(p-coumaroyl)-diglucoside-5-glucoside	Cya-3-pCo-Hex-Hex,-5-Hex	919.2	287.2	−50	−
Cyanidin 3-(feruloyl)-diglucoside-5-glucoside	Cya-3-Fe-Hex-Hex,-5-Hex	949.2	287.2	−50	−
Cyanidin 3-(feruloyl)(feruloyl)-diglucoside-5-glucoside	Cya-3-Fe-Hex-Fe-Hex,-5-Hex	1125.2	287.2	−50	−
Cyanidin 3-(glycopyranosyl-sinapoyl)-diglucoside-5-glucoside	Cya-3-Hex-Si-Hex-Hex,-5-Hex	1141.2	287.2	−50	−
Cyanidin 3-xylosylgalactoside	Cya-3-Pen-Hex	581.2	287.2	−20	−
Cyanidin 3-dioxalylglucoside	Cya-3-Ox-Ox-Hex	593.2	287.2	−20	−
Cyanidin 3-arabinosyl-rutinoside	Cya-3-Pen-Rut	727.2	287.2	−30	−
Cyanidin 3-xylosylglucosylgalactoside	Cya-3-Pen-Hex-Hex	743.2	287.2	−30	−
Cyanidin 3-glucosyl-rutinoside	Cya-3-Hex-Rut	757.2	287.2	−30	−
Cyanidin 3-(feruloyl)-glucoside-5-glucoside	Cya-3-Fe-Hex-5-Hex	787.2	287.2	−50	−
Cyanidin 3-xylosyl(coumaroylglucosyl)-galactoside	Cya-3-Pen-cMa-Hex-Hex	889.2	287.2	−50	−
Cyanidin 3-(caffeoyl)-diglucoside-5-glucoside	Cya-3-Ca-Hex-Hex,-5-Hex	935.2	287.2	−50	−
Cyanidin 3-(caffeoyl)(p-coumaroyl)-diglucosides-5-glucoside	Cya-3-Ca-pCo-Hex-Hex,-5-Hex	1081.2	287.2	−50	−
Cyanidin 3-(feruloyl)-triglucosides-5-glucoside	Cya-3-Fe-Hex-Hex-Hex,-5-Hex	1111.2	287.2	−50	−
Cyanidin 3-(sinapoyl)(sinapoyl)-diglucoside-5-(malonyl)glucoside	Cya-3-Si-Si-Hex-Hex,-5-Mn-Hex	1271.2	287.2	−50	−
Cyanidin 3-(feruloyl)(feruloyl)-triglucoside-5-glucoside	Cya-3-Fe-Fe-Hex-Hex-Hex,-5-Hex	1287.2	287.2	−50	−
Cyanidin 3-(feruloyl)(sinapoyl)-triglucoside-5-glucoside	Cya-3-Fe-Si-Hex-Hex-Hex,-5-Hex	1317.2	287.2	−50	−
***Delphinidin***					
Delphinidin 3-glucoside	Del-3-Hex	465.2	125.2	−60	+
Delphinidin 3-arabinoside	Del-3-Pen	435.2	125.2	−60	+
Delphinidin 3-rutinoside	Del-3-Rut	611.2	125.2	−70	+
Delphinidin 3,5-diglucoside	Del-3-Hex,-5-Hex	627.2	125.2	−70	−
Delphinidin 3-(6″-malonyl)-glucoside	Del-3-6”-Mn-Hex	551.2	303.2	−30	−
Delphinidin 3-(6″-acetyl)-glucoside	Del-3-6”-Ac-Hex	507.2	303.2	−30	−
Delphinidin 3-rutinoside-5-glucoside	Del-3-Rut,-5-Hex	773.2	303.2	−30	−
***Malvidin***					
Malvidin 3-glucoside	Mal-3-Hex	493.2	315.2	−60	+
Malvidin 3-arabinoside	Mal-3-Pen	463.2	315.2	−60	−
Malvidin 3-rutinoside	Mal-3-Rut	639.2	315.2	−70	−
Malvidin 3,5-diglucoside	Mal-3-Hex,-5-Hex	655.2	315.2	−70	−
Malvidin 3-(6″-malonyl)-glucoside	Mal-3-6”-Mn-Hex	579.2	331.2	−30	−
Malvidin 3-(6″-acetyl)-glucoside	Mal-3-6”-Ac-Hex	535.2	331.2	−30	−
Malvidin 3-(p-coumaroyl)-rutinoside-5-glucoside	Mal-3-pCo-Rut,-5-Hex	947.2	331.2	−30	−
***Pelargonidin***					
Pelargonidin 3-glucoside	Pel-3-Hex	433.2	141.2	−60	+
Pelargonidin 3-arabinoside	Pel-3-Pen	403.2	141.2	−60	−
Pelargonidin 3-rutinoside	Pel-3-Rut	579.3	141.2	−70	+
Pelargonidin 3,5-diglucoside	Pel-3-Hex,-5-Hex	595.2	141.2	−70	−
Pelargonidin 3-malonylglucoside	Pel-3-6”-Mn-Hex	519.2	271.2	−30	−
Pelargonidin 3-acetylglucoside	Pel-3-6”-Ac-Hex	475.2	271.2	−30	−
5-carboxypyrano-Pelargonidin-3-glucoside	5Carp-Pel-3-Hex	501.2	271.2	−30	−
Pelargonidin 3-malylglucoside	Pel-3-Ml-Hex	549.2	271.2	−30	−
Pelargonidin 3-sambubioside	Pel-3-Sam	565.2	271.2	−30	−
(Epi)afzelechin-(4–8)-Pelargonidin 3-glucoside	(Epi)afzelechin-(4–8)-Pel-3-Hex	705.2	271.2	−30	−
Catechin-(4–8)-Pelargonidin 3-glucoside	Catechin-(4–8)-Pel-3-Hex	721.2	271.2	−30	−
Pelargonidin 3-rutinoside-5-glucoside	Pel-3Rut,-5Hex	741.2	271.2	−30	−
(Epi)afzelechin-(4–8)-Pelargonidin 3-rutinoside	(Epi)afzelechin-(4–8)-Pel-3-Rut	851.2	271.2	−50	−
Pelargonidin 3-(p-coumaroyl)-rutinoside-5-glucoside	Pel-3pCo-Rut,-5-Hex	887.2	271.2	−50	−
Pelargonidin 3-(p-coumaroyl)-diglucoside-5-glucoside	Pel-3pCo-Hex-Hex,-5Hex	903.2	271.2	−50	−
Pelargonidin 3-(feruloyl)-rutinoside-5-glucoside	Pel-3-Fe-Rut,-5-Hex	917.2	271.2	−50	−
Pelargonidin 3-(feruloyl)diglucoside-5-glucoside	Pel-3-Fe-Hex-Hex,-5-Hex	933.2	271.2	−50	−
Pelargonidin 3-(p-coumaroyl)-diglucoside-5-(malonyl)-glucoside	Pel-3-pCo-Hex-Hex,-5-Mn-Hex	989.2	271.2	−50	−
Pelargonidin 3-(caffeoyl)-diglucoside-5-(malonyl)-glucoside	Pel-3-Ca-Hex-Hex,-5-Mn-Hex	1005.0	271.2	−50	−
Pelargonidin 3-(feruloyl)-diglucoside-5-(malonyl)-glucoside	Pel-3-Fe-Hex-Hex,-5-Mn-Hex	1019.2	271.2	−50	−
Pelargonidin 3-(feruloyl)(caffeoyl)-diglucoside-5-(malonyl)-glucoside	Pel-3-Fe-Ca-Hex-Hex,-5-Mn-Hex	1181.2	271.2	−50	−
Pelargonidin 3-(feruloyl)(feruloyl)-diglucoside-5-(malonyl)-glucoside	Pel-3-Fe-Fe-Hex-Hex,-5-Mn-Hex	1195.2	271.2	−50	−
***Peonidin***					
Peonidin 3-glucoside	Peo-3-Hex	463.2	201.2	−60	+
Peonidin 3-arabinoside	Peo-3-Pen	433.2	201.2	−60	−
Peonidin 3-rutinoside	Peo-3-Rut	609.2	201.2	−70	−
Peonidin 3,5-diglucoside	Peo-3-Hex,-5-Hex	625.2	201.2	−70	−
Peonidin 3-(malonyl)-glucoside	Peo-3-6”-Mn-Hex	549.2	301.2	−30	−
Peonidin 3-(6″-acetyl)-glucoside	Peo-3-6”Ac-Hex	505.2	301.2	−30	−
Peonidin 3-xylosylgalactoside	Peo-3-Pen-Hex	595.2	301.2	−30	−
Peonidin 3-(p-coumaroyl)-rutinoside-5-glucoside	Peo-3-pCo-Rut,-5-Hex	917.2	301.2	−50	−
Peonidin 3-xylosyl(feruloylglucosyl)-galactoside	Peo-3-Pen-Fe-Hex-Hex	933.2	301.2	−50	−
Peonidin 3-xylosyl(sinapoylglucosyl)-galactoside	Peo-3-Pen-Si-Hex-Hex	963.2	301.2	−50	−
***Petunidin***					
Petunidin 3-glucoside	Pet-3-Hex	479.2	216.2	−60	+
Petunidin 3-arabinoside	Pet-3-Pen	449.2	216.2	−60	−
Petunidin 3-rutinoside	Pet-3-Rut	625.2	216.2	−70	−
Petunidin 3,5-diglucoside	Pet-3-Hex,-5-Hex	641.2	216.2	−70	−
Petunidin 3-(6″-malonyl)-glucoside	Pet-3-6”-Mn-Hex	564.2	317.2	−30	−
Petunidin 3-(6″-acetyl)-glucoside	Pet-3-6”-Ac-Hex	521.2	317.2	−30	−
***Anthocyanin and flavonol***					
Cyanidin 3-glucoside & Kaemferol 3-glucoside	Cya-3-Hex & Kam-3-Hex	449.2	287.2	−20	+
Delphinidin 3-glucoside & Quercetin 3-glucoside	Del-3-Hex & Que-3-Hex	465.2	303.2	−20	+
Pelargonidin 3-glucoside & Apigenin 7-glucoside	Pel-3-Hex & Api-7-Hex	433.2	271.2	−20	+
Peonidin 3-glucoside & Chriserol 7-glucoside	Peo-3-Hex & Chr-7-Hex	463.2	301.2	−20	+
Petunidin 3-glucoside & Isorhamnetin 3-glucoside	Pet-3-Hex & Iso-3-Hex	479.2	317.2	−20	+
Delphinidin 3-arabinoside & Quercetin 3-arabinoside	Del-3-Pen & Que-3-Pen	435.2	303.2	−20	+−
Delphinidin 3-rutinoside & Quercetin 3-rutinoside	Del-3-Rut & Que-3-Rut	611.2	303.2	−30	+−
Delphinidin 3,5-diglucoside & Quercetin 3,5-diglucoside	Del-3-Hex,-5-Hex& Que-3-Hex,-5-Hex	627.2	303.2	−30	−
***Flavonol***					
Kaemferol 3-glucoside	Kam-3-Hex	449.2	85.2	−50	+
Quercetin 3-glucoside	Que-3-Hex	465.2	137.2	−50	+
Apigenin 7-glucoside	Api-7-Hex	433.2	153.2	−50	+
Chriserol 7-glucoside	Chr-7-Hex	463.2	153.2	−60	+
Isorhamnetin 3-glucoside	Iso-3-Hex	479.2	153.2	−60	+

a Polarity of all compounds was positive. The precursor ion of anthocyanin was [M]^+^, and the adduct ion of flavonol was [M + H]^+^.

b Presence (+) or absence (−) of the chemical standard for product ions selection. In the presence of the chemical standard, we confirmed the suitable product ions from the solution with 50% (v/v) ethanol by product ion scan mode. + − indicates that only delphinidin is present.

Next, we checked for the suitability of the SRM condition for plant samples (blueberry, apple, and onion). Specifically, we compared the intensities of product ions equivalent to Del-3-Hex or Que-3-Hex as the ion to be checked among differently colored crops. We used crude extracts to boost the intensity of the positive ions using a water/ethanol solvent, including formic acid. The skins of blueberries, apples, and onion bulbs were pared using a razor, dipped in the extraction solvent, and vortexed for 30 s. In this method of detecting anthocyanins in plants, the time and labor consumed in the pre-treatment steps are saved, unlike previous approaches, such as HPLC, which involve complicated purification steps.

**Figure 1:**
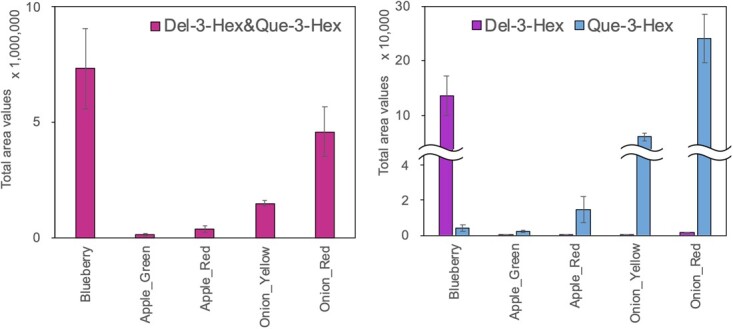
Detection of delphinidin 3-hexoside (Del-3-Hex) and quercetin 3-hexoside (Que-3-Hex) product ions in horticultural fruits and vegetables using PESI/MS/MS. Blueberry skin, apple skin, and onion bulb were pared using a razor and dipped in the extraction solvent. Each extract supernatant (10 μL) was placed on a plate for liquid and ionized using the PESI unit of DPiMS-8045. The spectral intensities of each product ion equivalent to anthocyanin or flavonol fragments or both were measured in selected reaction monitoring (SRM) transition mode, as described in Table 1. The intensities of product ions equivalent to Del-3-Hex and Que-3-Hex (*m/z* 303.2, collision energy (CE) −20 V; pink bars), Del-3-Hex alone (*m/z* 125.2, CE −60 V; purple bars), and Que-3-Hex alone (*m/z* 137.2, CE −50 V; blue bars) are presented. Total area values were calculated from the MS chromatogram during measuring time (0.3 min per compound). Vertical bars indicate standard errors (n = 3–4).

When comparing the profiles of product ions in this sample, the trend was found to be similar to that observed with the chemical standards. The product ion equivalent to Del-3-Hex at −60 V (*m/z* 125) was stably detected in blueberries (purple bar in Fig. 1). This is consistent with the results of previous studies, which the main anthocyanins in blueberry are delphinidin and malvidin [4, 30]. In contrast, the product ion equivalent to Del-3-Hex could hardly be detected in either green or red apple skins. A product ion comparable to Que-3-Hex (*m/z* 137) was detected stably in both the skins (blue bars in Fig. 1). In a previous study of apple [38], cyanidin was detected as the major anthocyanin in red skin, and delphinidin was not detected. Quercetin glycosides were detected in both green and red skins. Similar to apple, the product ion of Del-3-Hex was barely detected in onion, whereas that of Que-3-Hex was stably detected in both cultivars (Fig. 1). Regardless of the species and skin color, the product ion (*m/z* 303) was stably detected (pink bars in Fig. 1). Thus, we determined that the detected ion (*m/z* 303 at −20 V) in apple and onion was not mainly Del-3-Hex but Que-3-Hex. The two compounds could be distinguished by increasing the collision energy (*m/z* 125 at −60 V and *m/z* 137 at −50 V).

### Profiles of anthocyanins in horticultural crops using PESI/MS/MS

We utilized the SRM transition for 81 anthocyanins measurable for a total of 3 min (Table 1), and characterized the anthocyanin content profiles from the total area values calculated using absolute intensities. Crude extracts from edible parts in 16 species were used in the same manner as described in the previous section.

**Figure 2:**
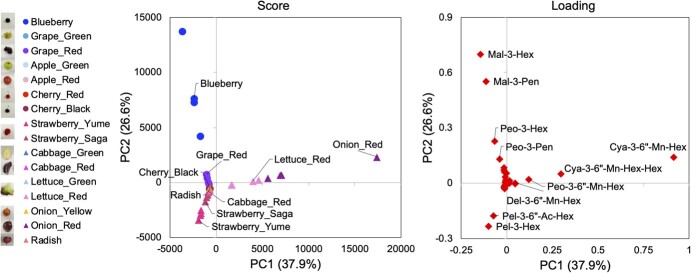
PCA score and loading plots for anthocyanins in horticultural crops using PESI/MS/MS. Blueberry, grape, apple, cherry, strawberry, and radish skins; cabbage and lettuce leaves; and onion bulbs were pared and dipped in the extraction solvent. Grape, apple, cherry, cabbage, and onion cultivars had different colors. Lettuce has two colors in one leaf. Two strawberry cultivars were analyzed, i.e. “Yumenoka” (Yume) and “Sagahonoka” (Saga). Each extract supernatant (10 μL) was placed on a plate for liquid and ionized using the PESI unit of DPiMS-8045. The intensities of the product ions and the total area values were calculated as described in Table 1 and Fig. 1. Score scatterplots show the PCA model of anthocyanins in the edible parts of horticultural fruits (circles) and vegetables (triangles) based on Pareto distribution (n = 3–4). Loading scatterplots were drawn from PCA analysis (rhombus, n = 80, anthocyanin peaks).

In principal component analysis (PCA), variance in PC1 score (37.9%) reflected the difference between red lettuce and red onion against other crops (Fig. 2). Cyanidins added to malonyl-hexose are the main contributors to this difference. This trend is consistent with previous reports on LC-based analysis [36, 37]. Variance in PC2 score (26.6%) reflected the difference between fruits (especially blueberries and grapes) and strawberries. These profiles were similar to those obtained by HPLC-ESI-MS/MS analysis [4, 7]. Most anthocyanins could not be detected in cultivars without a red color, including those of grape, apple, cabbage, lettuce, and onion (Supplementary Table S1). Thus, these cultivars did not vary in origin in PCA.

Most of the set product ions could be detected stably in cultivars with a red color during measurement time, which might be because the sample solution was stably supplied by repeatedly stirring the probe to a sufficient depth. Additionally, the SRM method was designed such that the scan rate per compound (1–2 ms) was faster than the ionization timing difference (150 ms), even when measuring compounds had different solvophobicities. This method provides exhaustive spectral intensities averaged for each anthocyanin over the entire analysis time. However, when detecting compounds with different polarities simultaneously, it is necessary to determine whether the ionization efficiencies of compounds are sequential and exhaustive [19].

Additionally, various anthocyanins, not reported in earlier studies, could be detected in many cultivars with a red color using the PESI/MS/MS method (Supplementary Table S1). We could not identify these compounds as precise anthocyanins in this study because of a lack of chemical standards. However, the PESI/MS/MS method may be effective as the first easy and rapid screening method for anthocyanin detection.

### A simpler assay for detecting anthocyanins using PESI/MS/MS; probe sampling

**Figure 3:**
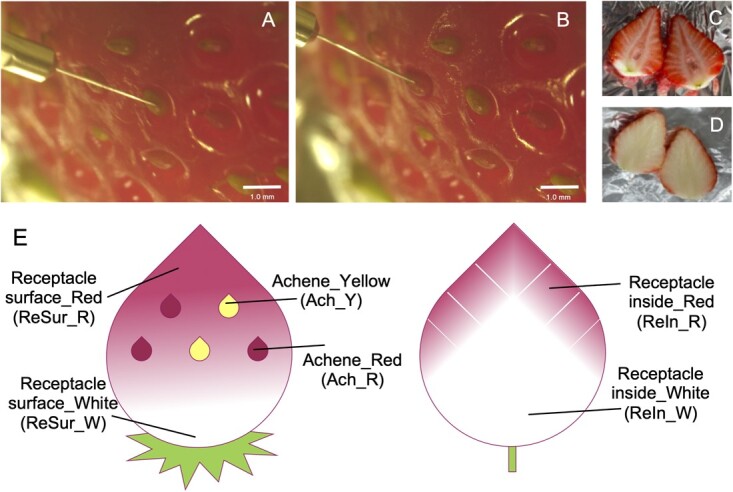
Strawberry fruit organs used for anthocyanin detection with probe sampling PESI/MS/MS. Achenes in ripe strawberry fruit (“Sagahonoka”; A, yellow; B, red) were measured using probes appurtenant to DPiMS-8045. Colors in the cut section of the receptacle were different between cultivars (C, “Yumenoka” and D, “Sagahonoka”). The tip of the probe was attached to each organ (E), the surface of achene (Ach), the surface of the receptacle (ReSur), and cut inside the receptacle (ReIn) in red (R) or non-colored (Y or W) areas in approximately one second. The tip was set on a PESI unit with 10 μL of the pure extraction solvent.

We used a more intuitive method of sticking a probe directly to the sample without pre-extraction; thus, we named this method “probe sampling”. We focused on strawberries as a measurement model. There are achenes and receptacles in strawberry fruit, and we visually distinguished the colors of these organs (Fig. 3). These shapes cannot be sampled using other ambient techniques, such as DART, which tests in an extensively open system environment. DESI can also perform fine sampling; however it requires imprinting on the TLC plate and is currently unsuitable for hard and small tissues such as achenes [12].

The degree of sample sticking to the probe tip was light only once, and the probe was placed immediately in the PESI unit. The extraction solvent was placed on a plate for liquid, and the adherent sample compounds at the tip were diluted and homogenized at the same time as ionization. The invasion depth into the solvent and the number of probe movements for repetitive samplings were the same as those in the crude extraction.

We used the same SRM transition in the crude extract samples, and 31 anthocyanins were detected in either strawberry organ. Of these, 29 overlapped with the results obtained using the crude strawberry extracts mentioned above. The signal intensities in the first segment were occasionally strong compared to those of the crude extraction, which then stabilized during the measurement period (data not shown), likely because the attached sample was initially over-ionized and then optimized upon dilution with the solution *via* vertical movement of the probe. Although these signal patterns may not be suitable for quantification, they are sufficiently stable for qualitative analysis.

**Figure 4:**
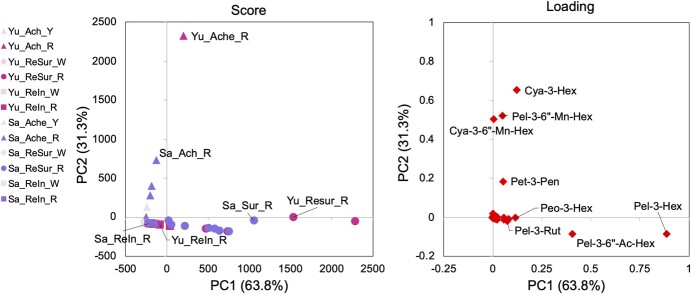
PCA score and loading plots for anthocyanins in strawberry organs using probe sampling PESI/MS/MS. Achene (Ach; triangle), surface (ReSur; circle), and inside (ReIn; square) of the receptacle in strawberry cultivars, i.e. “Yumenoka” (pink; Yu) and “Sagahonoka” (purple; Sa), in red (R) or non-colored (Y or W) areas were measured using the probes shown in Fig. 3. The pure extraction solvent was set on a plate for liquid, as shown in Fig. 1, and the probe was ionized using the PESI unit of DPiMS-8045. The intensities of the product ions and the total area values were calculated as described in Table 1 and Fig. 1. Score scatterplots show the PCA model of anthocyanins in strawberry organs based on Pareto distribution (n = 4–8). Loading scatterplots were drawn from PCA analysis (rhombus, n = 31, anthocyanin peaks).

In PCA, both variance in PC1 (63.8%) and PC2 score (31.3%) reflected the differences between organs (Fig. 4). Cyanidin 3-hexoside (Cya-3-Hex), cyanidin, and pelargonidin added to malonyl-hexoside (Cya-3-6”-Mn-Hex and Pel-3-6”-Mn-Hex) contributed to this difference. Pelargonidin (particularly Pel-3-Hex and Pel-3-6”-Ac-Hex) contributed to this difference between the surface and inside of the receptacle.

**Figure 5:**
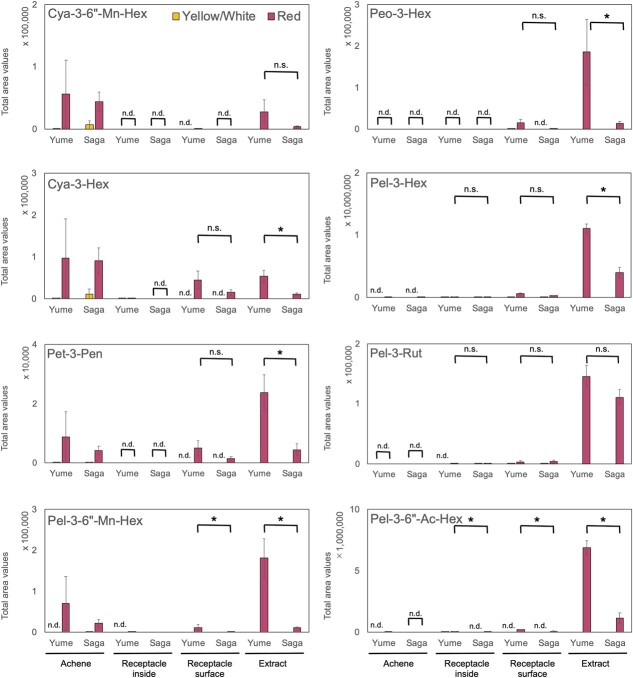
Comparisons of anthocyanins in strawberry organs using PESI/MS/MS. Strawberry organs of cultivars “Yumenoka” (Yume) and “Sagahonoka” (Saga) were collected using the probes described in Fig. 3. Each data point was extracted from Fig. 4 (achene, the inside, and surface of the receptacle) and Fig. 2 (extract, pre-treated sample). “n.d.” indicates “not detected” (i.e. total area value was zero). Asterisks indicate significant differences between the two red cultivars in the same organ (Wilcoxon test, *p* < 0.05). “n.s.” indicates “not significant”. Vertical bars indicate standard errors (n = 4–8).

We focused on the above-mentioned anthocyanins and compared the total area of the organs (Fig. 5). Cya-3-6”-Mn-Hex specifically accumulated in achenes compared to that in the receptacles. Cya-3-Hex and Pet-3-Pen mainly accumulated in the red-colored achene and on the receptacle surface. Contrastingly, Peo-3-Hex accumulated only on the surface of the receptacle. Pel-3-Hex, Pel-3-Rut, and Pel-3-6”-Ac-Hex were mainly present on the surface of the receptacle. These trends are generally consistent with those of earlier studies using MALDI-imaging MS and UPLC-qTOF-MS/MS, where Cya-3-Hex and Cya-3-6”-Mn-Hex were detected in achenes, and Cya-3-Hex, Pel-3-Hex, and Pel-3-Rut in the receptacle in “Tochiotome”, “Seascape” and “Herut” [39–41]. The intensity of Pel-3-6”-Mn-Hex in achene and receptacle was not consistent in “Herut” [41], and accurate quantitative comparisons between different organs in the probe sampling are currently not feasible because each cell state is different. Additionally, in a few cases, the simplistic handling of probe sampling may be a factor in data variability (Fig. 5). Thus, this method can be regarded as a qualitative analytical method.

Based on the difference of organs, we considered only the cultivar-specific differences within the same organ category for the two cultivars, i.e. “Yumenoka” and “Sagahonoka”. The inside of the receptacle in “Yumenoka” was widely reddish, and that of the “Sagahonoka” receptacle was reddish only at the edge of the fruit (Figs. 3C and 3D). Pelargonidins were detected mainly on the surface of the receptacle, with the total area of Pel-3-6”-Ac-Hex in “Yumenoka” being significantly higher than that in “Sagahonoka” (Fig. 5). These trends were similar, even the inside of receptacle obtained by probe sampling and the extract of the pre-treated sample.

Several methods similar to probe sampling have been reported, including dipping PESI/MS (dPESI/MS) [42], remote sampling in sheath-flow PESI (sfPESI) and adjustable sfPESI (ad-sfPESI) [17, 43]. dPESI/MS is a different control setting for probe ionization than in probe sampling. Compared to the shallow invasion depth (<0.5–1 mm) and several shots in dPESI/MS, PESI/MS/MS was set at a depth of 2 mm which was deeper, and more repetitive samplings were conducted. This condition stabilized the signal profile and resulted in more continuous ionization and detection in the SRM mode. In sfPESI and ad-sfPESI, the structure of the probes was more precise, but more complicated than that in PESI/MS/MS. Additionally, water-soluble compounds could not be detected using sfPESI in the remote sampling of apple skin. The *m/z* intensities corresponding to the anthocyanin fragments could not be observed. However, in probe sampling of strawberry skin (receptacle and achene) by PESI/MS/MS, hydrophilic anthocyanins were stably detected even with one probe stick and pure solvent alone. A general comparison was not feasible because the measurement conditions and samples differed, but the difference in solvents, i.e. water/methanol in sfPESI and water/ethanol in PESI/MS/MS, might have contributed to the ionization efficiencies at plant surfaces. Additionally, regarding the detectors, previous ambient approaches mainly used single quadrupole or Orbitrap mass spectrometer [11, 12, 19, 43], and MS/MS spectra have not yet been exhibited. In other instruments [39–41], sample pre-treatment, such as matrix application in MALDI-imaging, was complicated and qualitative. LC–MS/MS requires large amount of samples for separation using HPLC. In that respect, PESI/MS/MS could be effective as the first screening to detect anthocyanins in horticulture crops at the micro-area scale, with the combination of simplified probe sampling and avoiding the use of overly large scale MS instruments.

As we confirmed that partially independent metabolic pathways are locally regulated in the fruit using probe sampling (Figs. 3 and 4), it is possible to clarify temporal and spatial metabolic changes that are difficult with conventional analysis of the whole fruit or complicated analysis operations. In strawberries, the differences observed between achene and receptacle would provide insights to accelerate the elucidation of the physiological mechanism and breeding of new cultivars with independent colors in these organs [44]. A combinatorial approach using microscopy and probes may help elucidate mechanisms underlying the differences in metabolite accumulation at the cellular level [15].

In conclusion, we established a high-throughput analysis method for measuring 81 anthocyanins in approximately 3 min using PESI/MS/MS. The number of measurable molecular species was increased by devising the method that enables specific and long-term measurements. In addition to extracting solutions, probe sampling can qualitatively detect anthocyanins at a micro-scale. Proper implementation of these methods in research using plant materials is crucial. Measurable compounds other than anthocyanins and measurable plant species, including crops and ornamental flowers, need to be amplified for future research. For research on the metabolism of animals, such as mice, pipelines were constructed using PESI/MS/MS for high-throughput metabolome and statistical analysis [22, 24]. PESI/MS/MS can be a powerful tool for breed selection and quality assessment in the field of horticultural science, which would help detect the indicators of physiological responses in cultivation field, such as stress response markers. The portable probes allow the sample to be remote from the front of the MS instrument. Consequently, it would be possible to understand crop characteristics of several remotely located fields simultaneously if the transportation conditions of the sampled probes are established.

## Materials and methods

### Plant materials

We purchased horticultural fruits and vegetables from local markets in Nagoya City, including blueberry (*Vaccinium corymbosum*), grape (*Vitis vinifera*, green and red skin), apple (*Malus* × *domestica*, green and red skin), cherry (*Prunus avium*, red and black skin), strawberry (*Fragaria* × *ananassa*, “Yumenoka” and “Sagahonoka”), cabbage (*Brassica oleracea var. capitata*, green and red leaves), lettuce (*Lactuca sativa* var. crispa, green and red leaves within a plant), onion (*Allium cepa*, yellow and red bulbs), and radish (*Raphanus sativus* var. sativus). The edible parts (skin, leaf, and bulb) were pared using a razor and translated into micro-tubes. After measuring the fresh weights of these pieces, tubes were immediately frozen in liquid nitrogen and stored at −80°C until use.

### Reagents

We purchased the standards of anthocyanins and flavonols from several companies, including Nagara Science Co., Ltd. (Gifu, Japan; cyanidin 3-glucoside chloride, cyanidin 3-rutinoside chloride, delphinidin 3-glucoside chloride, and delphinidin 3-rutinoside chloride), Extrasynthese (Lyon, France; pelargonidin-3-O-glucoside chloride, pelargonidin-3-O-rutinoside chloride, and cyanidin-3,5-di-O-glucoside chloride), TOKIWA Phytochemical Co., Ltd. (Chiba, Japan: delphinidin 3-arabinoside, malvidin 3-glucoside chloride, peonidin 3-glucoside chloride, and petunidin 3-glucoside chloride), Santa Cruz Biotechnology Inc. (Heidelberg, Germany; kaempferol-3-glucoside), Sigma-Aldrich (Saint Louis, MO, USA; apigenin 7-glucoside and quercetin 3-β-D-glucoside), and ChemFaces Biochemical Co., Ltd. (Hubei, China; isorhamnetin-3-O-β-D-glucoside and chrysoeriol-7-O-glucoside). Solid reagents were dissolved in 99 or 50% (v/v) ethanol and stored at −80°C until use. The stock solutions were diluted with 50% (v/v) ethanol (1–100 ppm) and used for optimizing conditions.

### Analysis conditions using PESI/MS/MS

A DPiMS-8045 (LCMS-8045 tandem mass spectrometer with probe electrospray ion source; Shimadzu Corporation, Kyoto, Japan) and PESI MS solution (ver 2.0.8) were used for analysis. Frozen sample pieces, except cabbages, were dipped in a volume of the extraction solvent (0.1% formic acid and 49.95% ethanol (v/v)) ten times their weight, and vortexed for 30 s. The cabbage pieces were dipped a volume of the extraction solvent two times their weight. The supernatant (10 μL) was placed on a plate for liquid (Shimadzu Corporation), and ionized using a PESI unit of DPiMS-8045. We used a PESI probe with a tip diameter of approximately 700 nm and a body diameter of nearly 0.14 mm (Shimadzu Corporation). For probe sampling, the tip of the probe was attached to the organs of ripe raw strawberry fruits at room temperature (approximately 20°C) for one second. Subsequently, 10 μL of pure extraction solvent was placed on a plate for liquid and ionized.

The product ion scan modes were set as follows: scan range from 100 to precursor ion +10 (*m/z*), collision energy to the maximum from −10 to −70 V, scan speed to 5000 u/s, and measurement time to 60 s. The details of SRM mode for each analysis, such as the SRM transition and collision energy, are listed in Table 1. The dwell and pause times for SRM transition were 1 ms each. We set up ten parallel segments in the SRM transition mode, with each measurement time set to 0.3 min. There were 92 total measuring segments, i.e. the measurement time per sample was 3 min. The frequency of the PESI probe was 11.47 Hz, and the ionization and sampling times were 150 and 30 ms, respectively. The probe was at an upper position during ionization, and at a lower position during sampling. Repetitive samplings were performed 60 times per 0.3 min. The invasion depth of the probe in the sample solution was approximately 2 mm. The applied voltages for the PESI positive modes were 2.5 kV.

### Statistical analyses

The calculation software, LabSolutions (ver. 5.86, Shimadzu Corporation) was used to confirm each anthocyanin peak in the MS chromatogram and calculate the total area values. Principal component analysis (PCA) was performed using Statistics in Microsoft Excel [45]. Other statistical analyses were performed using JMP 13 (SAS Institute, Inc., Cary, NC, USA). The data represent mean values ± standard error of three to eight biological replicates.

## Acknowledgments

This work was supported by the Japan Society for the Promotion of Science KAKENHI [grant numbers 20J00948, 20K20372, 21K19111, and 21H02184]. We are grateful to Drs. Kanako Sekimoto (Yokohama City University), Hidekazu Saiki, Eishi Imoto (Shimadzu Corporation), Yoshinori Fujimura (Kyushu University), Kazuyoshi Terasaka (Nagoya City University), Hidetoshi Ikegami (Fukuoka Agriculture and Forestry Research Center), and Kenta Tsunekawa (Aichi Agricultural Research Center) for helpful discussion.

## Author Contributions

K.S. and M.I. conceived and designed the study; K.Z. organized the system construction for the PESI/MS/MS method; M.I. and I.Y. performed the analysis with the support of K.Z.; M.I., I.Y. and K.S. analyzed data; K.Z. S.O., S.M. and A.O. contributed to data analysis; M.I. and K.S. wrote and revised the manuscript accordingly; All authors have read and approved the manuscript.

## Data availability

All data supporting the findings in this study are included in this article and supplementary materials.

## Conflict of interest statement

The authors declare that they have no conflict of interest.

## Supplementary Data

Supplementary data is available at *Horticulture Research* online.

## Supplementary Material

Web_Material_uhad039
