# Supplementary material for: High-throughput analysis of anthocyanins in horticultural crops using probe electrospray ionization tandem mass spectrometry (PESI/MS/MS)
Source: Hortic Res. 2023 Feb 28;10(4):uhad039. doi: 10.1093/hr/uhad039 (PMC10111199; doi:10.1093/hr/uhad039)
Supplement: Web_Material_uhad039 [file web_material_uhad039.zip › PESI_SupFig1.docx]

**Supplemental Figure 1.** MS spectra of chemical standards (A: delphinidin 3-glucoside and B: quercetin 3-glucoside) in the product ion scan mode using PESI/MS/MS.

Each standard solution (1 ppm) was diluted with 50 % (v/v) ethanol, and 10 µL set on a plate for liquid and ionized by PESI unit of DPiMS-8045. The intensities of the product ions equivalent to these fragments were measured in product ion scan mode as follows: positive polarity, scan range from 100 to 475 (*m/z*), scan speed 5,000 u/s, and collision energy −60 V (A) or −50 V (B). The precursor ion (*m/z*) was set at 465.2 both. The MS spectra were generated using LabSolutions. Absolute intensities integrated MS chromatogram during the measurement time (60 s). Specific detected ions (yellow arrows) were selected in the SRM mode, as described in Table 1. Images of the chemical structures were obtained from MassBank (accession numbers: PR311137 and PR302835).
